# PPE59 antibodies in tuberculous patients and potential use for diagnosis when assayed with other rapid biomarkers

**DOI:** 10.1590/0074-02760230183

**Published:** 2024-09-16

**Authors:** Ana Carla de Paulo Mulinari, Isabela Gama Sardella, Vania Maria C da Silva, Alberto Matteelli, Anna Cristina C Carvalho, Maria Helena Féres Saad

**Affiliations:** 1 Fundação Oswaldo Cruz-Fiocruz, Instituto Oswaldo Cruz, Laboratório de Microbiologia Celular, Rio de Janeiro, RJ, Brasil; 2 Universidade Federal do Rio de Janeiro, Faculdade de Medicina, Rio de Janeiro, RJ, Brasil; 3 Università degli Studi di Brescia, Clinic of Infectious and Tropical Diseases, Spedali Civili di Brescia, Italy; 4 Fundação Oswaldo Cruz-Fiocruz, Instituto Oswaldo Cruz, Laboratório de Inovações em Terapias, Educação e Bioprodutos, Rio de Janeiro, RJ, Brasil

**Keywords:** Mycobacterium tuberculosis, tuberculosis, 16kDa, serology, PPE59 (*rv3429*)

## Abstract

**BACKGROUND:**

PPE 59, which is absent from bacillus Calmette Guérin (BCG) strains, seems to induce a humoral immune response in patients with tuberculosis (TB). Additional studies are needed to better evaluate this protein in immune response to tuberculosis.

**OBJECTIVES:**

To evaluate the response of antibodies to PPE59 in TB individuals, its combination with IgG response to other, previously tested mycobacterial antigens (Ag) and with sputum smear microbiology (SM) results.

**METHODS:**

We have cloned and expressed the *rv3429* gene that encodes PPE59, then IgG, IgM, and IgA against PPE59 antigens measured by enzyme-linked immunosorbent assay (ELISA) in 212 sera samples obtained from the following subject cohorts: TB residents from Italy (79) and in Brazil (52); and an all-Brazilian cohort of 55 patients with other respiratory disorders; 10 patients infected with non-tuberculous mycobacteria, and 16 asymptomatic subjects. Drawing on results from a previous study^(17)^ of serum samples from Brazilian subjects tested for IgG by ELISA against mycobacterial antigens ESAT-6, 16kDa, MT10.3, MPT-64 and 38kDa, the results were analysed in combination with those of the PPE59 and SM tests.

**FINDINGS:**

Keeping the specificity rate at 97%, the overall PPE59 IgA sensitivity was 42.7%, while IgG and IgM showed lower performance (p < 0.0001). Combining PPE59 IgA/16kDa IgG results increased sensitivity to 71%, and even higher rates when the results were combined with SM results (86.5%, p = 0.001), at 88.9% specificity. Positive IgA was associated with pulmonary image alterations of high TB probability (p < 0.05).

**MAIN CONCLUSIONS:**

Tests with TB patients found a moderate frequency of positivity for PPE59 IgA. However, the higher level of sensitivity attained in combination with PPE59 IgA/16kDa IgG/SM results unheard of before, although imperfect, suggests that this may be a potential additional tool for rapid detection of TB in low-resource areas.

Despite the availability of effective treatment for tuberculosis (TB), a disease caused by *Mycobacterium tuberculosis* bacilli (Mtb), it remains among the twenty top causes of death globally and the leading cause of mortality from a single infectious agent, surpassing that of human immunodeficiency virus/acquired immunodeficiency syndrome HIV/AIDS.^1^ Active TB diagnosis relies on initial clinical suspicion and radiographic evidence, and subsequently on laboratorial confirmation.^2^ Delays in TB diagnosis can deter early treatment, playing a role in the continued spread of TB in the community.^3^ The longest-existing immune-based tests for TB diagnosis, *i.e.*, tuberculin skin test (TST) and serology, although relatively cheap, provide low specificity rates due to cross-reactivity with antigens present in both bacillus Calmette Guérin (BCG) vaccine and non-tuberculous mycobacteria (NTM) species.^4^ Serology holds out potential to be used at point-of-care and field work, and is particularly useful when working with special demographics, such as immigrants, indigenous populations, and homeless people.^5^ ^,^ ^6^

Proteins in the proline-proline-glutamate (PPE) family have yet to be studied more extensively. Research has suggested they play a role in several aspects of pathogenesis, including bacterial attachment to host cells, immunomodulation, and ability to persist in granuloma.^7^ PPE proteins play a key role in generating antigenic variations.^8^ ^,^ ^9^ Other authors have demonstrated that PPE41 induced B-cell immune responses in tests using a panel of human sera from patients with active TB and relapsed patients with extrapulmonary TB.^10^ ^,^ ^11^ PPE Rv1168c was described as having stronger specific immunoreactivity in the sera of patients with active TB compared to PPD, ESAT-6, and hsp60.^12^ On the other hand, anti-PPE55 antibody differential detection elicited from subclinical TB infection in animal subjects, as well as their PPE-C peptide in HIV-negative humans with clinical TB months prior to TB manifestation on HIV-positive cases, suggested it has been useful for differentiating between TB statuses.^13^ Moreover, *rv3429c* genes that encode PPE59 proteins have shown low C-terminal homology to other mycobacterial species and elicited low T-cell immune responses.^14^ Although the authors of the study have stated that it induced a humoral immune response in TB patients, no additional research on this response has been reported. The gene *rv3429c* belonged the region of difference (RD)-11, region absent in *M. bovis* and most mycobacterial species.^14^ ^,^ ^15^ ^,^ ^16^

Thus, our study has aimed to clone and express the *rv3429* gene that encodes PPE59 and evaluate the immunoreactivity of IgG, IgM and IgA isotypes against PPE 59 antigen (Ag) in sera from Brazilian and Italian TB patients and their correlation with their clinical and demographic data.

Additionally, in a past study,^17^ we have found low IgG ELISA reactivity (< 37%) to several mycobacterial antigens (ESAT-6, 16kDa, MT10.3, MPT-64 and 38kDa) in TB patients. However, diagnosis of TB may require other rapid and affordable tools to improve diagnostic efficiency, especially in low-resource, TB-endemic areas. Therefore, we have drawn on the findings of that prior study using the same serum samples from Brazilian subjects used herein,^17^ for a combined analysis of the results obtained from PPE59 and sputum smear microscopy (SM), which remains the flagship method for TB diagnosis in many TB-endemic areas.

## MATERIALS AND METHODS

*Cloning, expression and purification of recombinant PPE 59 protein* - The *rv3429* gene sequence (Gene ID: 887630) encoding the PPE 59 protein was generated by polymerase chain reaction (PCR) from the genomic DNA of H37Rv using upstream 5′GCG^GATCCATGCATCCAATGATA3′ and downstream 5′CA^AGCTTCTACCCGCCCCCGCCCCCGTA3′ primers, comprising BamHI and HindIII restriction endonucleases, respectively. The standard conditions for thermal cycle amplification were as follows: 94ºC for 5 min; 25 cycles of 1 min each of 94ºC, 60ºC, and 72ºC; final cycle of 72ºC for 5 min. The cloning, expression and purification steps were completed as described previously, with minor modifications at protein purification.^18^ Briefly, the transformed expression system containing the pQE80L-*rv3429* was better induced by 1 mM isopropyl-beta-D-thiogalactopyranoside (IPTG) at 37ºC for 4 h. Due to the presence of recombinant proteins in the insoluble fraction, the His-tag fusion protein was purified under denaturing conditions with 8M urea lysis buffer. The overexpressed recombinant proteins were confirmed by probing with anti-His-tag monoclonal antibody (Invitrogen, USA) and 15% SDS-PAGE (sodium dodecyl sulfate-polyacrylamide gel electrophoresis) analysed. Solubilised protein was loaded into HisLink Protein Purification Resin (Promega, USA) and eluted at different concentrations (5 mM, 20 mM, 100 mM and 250 mM) of imidazole (Qiagen of Brazil) buffer and of urea (8 M and 6 M). The release of the expected target protein was analysed by Western blot with anti-His-tag monoclonal antibodies. Target protein concentration was measured by Coomassie Protein Assay (Thermo Scientific, USA).

*Clinical samples* - In order to test IgG, IgM, and IgA antibodies against PPE59, 212 sera were included in our survey. All patients were recruited from a prior study^17^ and had their diagnosis done at a primary health care centre (Centro de Saúde Municipal Heitor Beltrão) in Rio de Janeiro, an endemic area for TB in Brazil. In group I, consisting of 133 samples from Brazilian subjects (BR), patients were categorised according to their final diagnosis, based on ATS/IUATLD criteria.^19^ pTB^BR^ (pulmonary tuberculosis, n = 52): symptomatic respiratory patients presenting positive SM for acid-fast bacilli (AFB) and/or positive Mtb culture performed with solid Lowenstein-Jensen media, or with negative SM, negative or no culture results but chest X-Ray (CXR) improvement after two months of first-line multi-drug anti-tuberculosis therapy. Other respiratory disorders (ORD, n = 55): symptomatic respiratory patients with negative SM, TST ≥ 10mm, or TST <10 mm, and/or other pulmonary conditions, such as asthma, bronchitis, sinusitis, pneumonia, Wegener’s granulomatosis, chronic obstructive pulmonary disease (COPD) and cancer, or patients with a history of TB contact. Mtb culture was required for all patients with chronic pulmonary disorders. All patients were followed for one more year to confirm the final diagnosis. We included 16 asymptomatic respiratory donors (HD^BR^), of which eight were TST-positive with no known history of, or contact with, TB. Additionally, we assayed 10 sera from Brazilian patients with pulmonary infection by non-tuberculous mycobacteria (NTM), such as *M. avium*, *M. avium- intracellulare*, *M. fortuitum*, *M. abscessus*, *M. kansasii* or *M. szulgai*. All cases were confirmed by culture, and sera kindly donated by Dr R Olmo (Leprosy Laboratory, Fiocruz). Group II consisted of 79 TB patients born (n = 12) or immigrants to Italy (IT), of which 54 were pTB^IT^ and 25 were extrapulmonary TB (eTB^IT^) patients. Immigrants enrolled were from Africa (n = 22), including 10 from Senegal, seven from Morocco, and one from each of Gambia, Ghana, Kenya, Liberia, and Nigeria; Asia (n = 34), including 20 from India, 12 from Pakistan, and two from the Philippines; and 10 from Europe (four Romanian, three Moldavian, two from Kosovo and one Russian). Body sites for eTB^IT^ included lymph node (n = 10), bone (n = 3), vertebra (n = 2), pleura, intestine, adrenal gland, or epididymis (one, each), and multiple tissue (n = 6). Mtb culture was performed by Bactec MGIT. These patients had TB diagnosed and treated at the Infectious and Tropical Diseases Clinic of Azienda Ospedaliera Spedali Civili di Brescia, Italy, in the period from 2011 to 2012.

The TB^IT^ patients were already on TB treatment with first-line TB drugs before blood withdrawal, whereas Brazilians were free of treatment at the time.

All individuals were aged ≥ 18 years and HIV-negative. All TB diagnoses were based on clinical, radiographic, and microbiological methods. SM was performed using the hot Ziehl-Neelsen method and semi-quantitative AFB was analysed as positive or negative. CXR changes were classified for low (normal or suggestive of pulmonary disease), intermediate (infiltrates) and high probability for TB (infiltrates and/or cavities).^19^ Only TB^IT^ patients were tested by interferon-gamma release assay (IGRA) using Quantiferon Gold Tube^®^ for *Mtb* infection detection.

The study was performed according to the Code of Ethics of the World Medical Association (Declaration of Helsinki) and approved by the Fiocruz Research Ethics Committee (No. 560-10) and the Clinical Ethics Committee di Malattie Infettive - Spedali Civili of the Institute of Infectious and Tropical Diseases of the University of Brescia, Italy (N. 196/03-11).

*Enzyme-linked immunosorbent assay (ELISA)* - In order to analyse the individual immune response from IgG, IgM and IgA to the PPE59 protein, ELISA was standardised for each isotype using a pool of sera from 10 positive SM pTB^BR^ and 16 HD^BR^ (half-positive TST) subjects. The tests were performed in 96-well, flat-bottom microplates (Nunc, USA) coated with antigen at 0.5 and 1.0 µg/mL prepared in carbonate/bicarbonate coating buffer, pH = 9.6, and incubated at 37ºC for 2 h. To prevent non-specific binding, we used a 5% solution of bovine serum albumin (BSA, fraction V, Sigma, Saint Louis, MO, USA) in phosphate-buffered saline containing 0.01% Tween 20 (PBSt). The test was conducted as described in a previous study^20^ with minor modifications, including 1:1000 diluted HRP-conjugate goat anti-human IgG and IgM (Thermo Fisher Pierce, USA) and 1:2500 diluted anti-human IgA. Serum aliquots did not undergo freezer-thaw cycling before testing. Individual response for all samples was blind tested.

Building on previous tests on the Brazilian sera samples for ELISA IgG with the mycobacterial ESAT-6, 16 kDa, MT10.3, MPT-64 and 38 kDa antigens,^17^ the results were analysed in combination with IgA and IgG PPE59 reactivity.

*Data analysis* - Diagnostic codes were opened at the end of all tests, and HD^BR^ and ORD^BR^ used as reference control groups for receiver operating characteristic curve analysis (ROC), setting the cut-off value for sensitivity and specificity calculation. Antibody levels were analysed as a function of medium absorbance and minimum and maximum. GraphPad Prism 7 software (San Diego, CA, USA) was used for statistical analysis. As the data was not normally distributed, non-parametric Kruskal Wallis was performed to analyse signiﬁcant differences in all groups and Mann-Whitney test was used for pairwise comparisons, whereas Fisher exact test and chi-square test with Yates’s correction were used to evaluate the significance of the difference between the numbers of positives in different serology tests. Accuracy was calculated over the total of true positives and true negatives, using the formula (true positive + true negative)/total number of populations studied, based on the following indicator ranges: values between 0 and 40% (low accuracy), 50-70% (moderate accuracy) and 80-100% (high accuracy). Odds ratios (OR) and logistic regression were used in uni- and multivariate analysis, with p values ≤ 0.05 considered significant with their respective 95% confidence intervals. SPSS Statistics 18 (IBM Corporation) also used for data analysis.

## RESULTS

*Characteristics of study subjects* - The clinical, epidemiological and laboratory characteristics of the adult participants are shown in Table I. In group I, HD participants were younger than the other subjects (p = 0.01) and most of the patients were male for both groups. Moreover, the group included a significant number of alcohol users (p = 0.004), unlike group II. A majority (88.6%) of group II patients (compared to 65.3% in group I) were diagnosed through positive SM and/or culture; only seven pTB^BR^ and five pTB^IT^ had probable TB. Approximately 60% of the pTB^BR^ sample showed CXR findings of high probability for TB, whereas among Italians/immigrants, intermediate changes predominated (p = 0.03). A large portion of group I subjects had a BCG scar among HD (100%), decreasing toward ORD (49%) and pTB (38%), while the occurrence of positive TST was the reverse: 50.0%, 61.8%, and 78.8% respectively. TST information was not available for group II, but Quantiferon^®^ tests were performed in 53% of subjects in the group, returning positive results for 44% (eTB^IT^) and 48% (pTB^IT^) of them. Most TB^IT^ patients (84.8%) were already on treatment with first-line TB drugs, while TB^BR^ were free of treatment at blood withdrawal.

**TABLE I:** Characteristics of the study subjects

	Study group I (n = 133) (%)				Study group II (n = 79) (%)	
	pTB^BR^ n = 52	ORD^BR^ n = 55	NTM^BR^ n = 10	HD^BR^ n = 16	pTB^IT^ n = 54	eTB^IT^ n = 25
Age (M ± SD)	(45.7 ± 18.2)	(49.1 ± 18.8)	-	(33.8 ± 11.3)^1^	(39.4 ± 16,9)	(41.9 ± 15.6)
Male	35 (67.0)	25 (45.0)	4 (40)	6 (37.5)	42 (78.0)	12 (48.0)
Alcohol user	20 (38.4)^2^	8 (14.5)	-	0	7 (12.9)	0
No information	1 (1.9)	0	10 (100)	0	19 (35.1)	3 (12.0)
Tobacco user	22 (42.3)	12 (21.8)	-	0	14 (25.9)	6 (24.0)
No information	1 (1.9)	0	10 (100)	0	20 (37.0)	3 (12.0)
Presence of BCG scar	20 (38.4)	27 (49.0)	-	16 (100.0)	-	-
TST and/or IGRA						
Positive	41 (78.8)	34 (61.8)	-	8 (50.0)	26 (48.1)	11 (44.0)
Not done	7 (13.4)	0	-	-	24 (44.4)	13 (52.0)
AFB-SM and/or culture						
Positive	34 (65.3)	0	10	-	49 (90.7)	21 (84.0)
Not done	7 (13.4)	11 (20.0)	0	16 (100.0)	3 (5.5)	2 (8.0)
CXR probability for TB						
High	31 (59.6)^2^	1 (1.81)	-	-	20 (37.0)	0 (0.0)
Intermediate	10 (19.2)	2 (3.63)	-	-	25 (46.2)^3^	6 (24)
Low/Normal	10 (19.2)	52 (94.5)	-	-	1 (1.85)	17 (68)
Not done	1 (1.92)	0 (0.0)	-	16 (100.0)	8 (14.8)	2 (8.0)
Not available			10 (100)			
Anti-TB therapy						
Free	52 (100.0)	55 (100.0)	-	NA	12 (22.0)	-
Treated	-	-	-	-	42 (78.0)	25 (100.0)

M ± SD: mean ± standard deviation; pTB or eTB: pulmonary or extrapulmonary tuberculosis; ORD: other respiratory disorder; NTM: infection by non-tuberculosis mycobacteria; HD: asymptomatic donor; BR: Brazilian; IT: Italian/ immigrants in Italy; TST: tuberculin skin test; IGRA: interferon gamma released assay (only performed on immigrants); AFB-SM: semi-quantitative analysis of acid-fast bacilli by sputum smear microscopy; CXR: chest X-ray; 1 p = 0.01; 2, 3 p = 0.03.

**Fig. 1:**
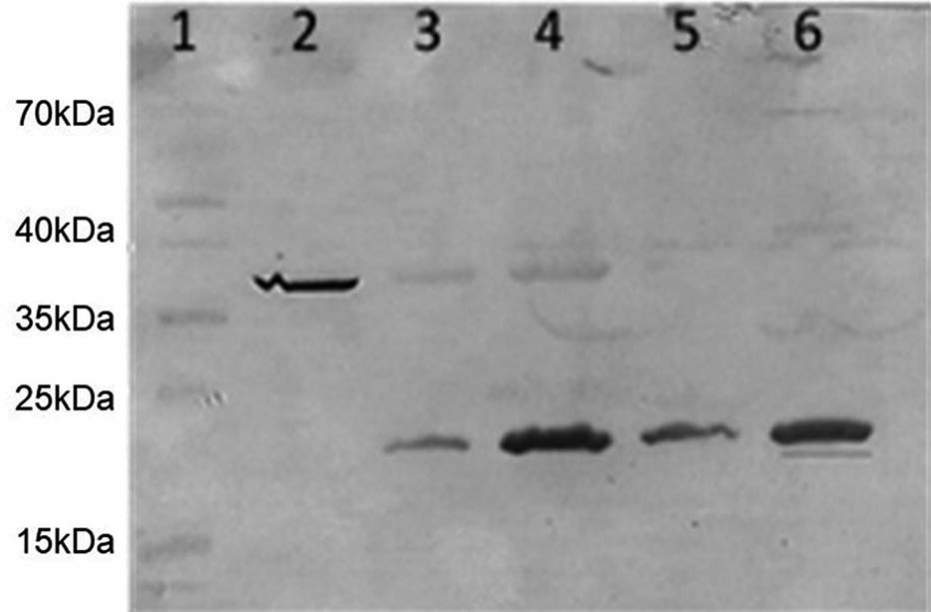
western blot of recombinant PP59 protein. Purification process under denaturing conditions, incubated with mouse IgG anti-His. Lane 1: pre-stained protein molecular ladder; lane 2: positive control; line 3: overnight pellet; line 4: supernatant; lines 5 (used in the study) and 6: eluates of PPE59 (MW = 21kDa).

*Cloning, expression, and purification of PPE 59 protein* - The 534bp of *rv3429* successfully cloned into pQE80L, and most of the expressed PPE59 protein was present in the inclusion bodies of the pellet being purified under denaturing conditions with 8 M urea. The release of the target protein from the Ni-resin column with expected molecular weight (21 kDa) occurred in the eluate containing 6 M urea and 100 mM imidazole, and the concentration of the expressed protein was high, 700 µg/mL). The protein proved to be stable under stored condition (4ºC or 8ºC), and the same batch was used for all immunological tests (Fig. 1A, line 5).

**Fig. 2:**
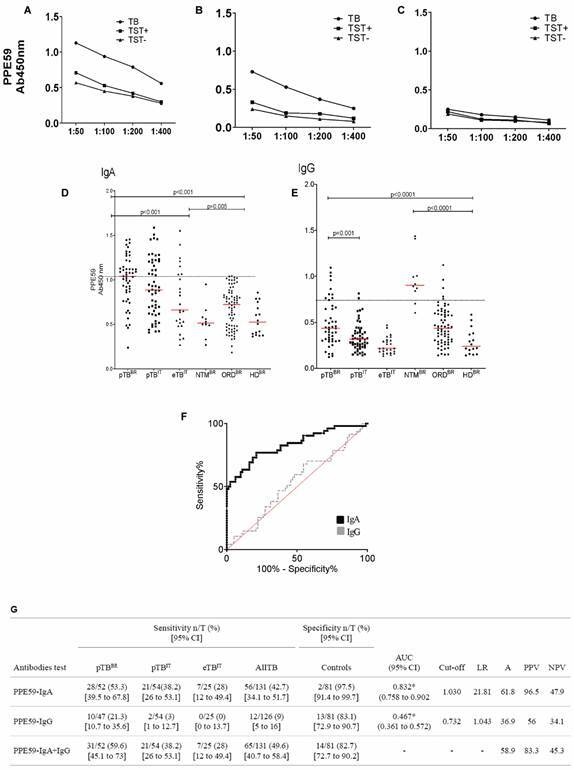
humoral responses by IgA, IgG and IgM enzyme-linked immunosorbent assay (ELISA) PPE59. The IgA (A), IgG (B) and IgM (C) -ELISA standardisation with recombinant PPE 59. Pools of sera from 20 Brazilian individuals with pulmonary tuberculosis (•) and eight asymptomatic donors with positive (■) or negative (▲) TST, respectively, at serial dilution 1:50 to 1:400. Distribution of IgA (D) and IgG (E) response to PPE59 in sera of Brazilians (BR) and Italians/immigrants (IT). Dotted line: cut-off point at 1.030 for PPE59-IgA and 0.732 for PPE59-IgG. NTM: non-tuberculous mycobacteria pulmonary infection. ORD: other pulmonary disorders. HD: asymptomatic respiratory individual with no history or known contact with tuberculosis. (F/G) Evaluation of area under the curve (AUC) of the receiver operating characteristic (ROC) PPE59-IgA/IgG and (G) immune response qualitative data. AUC was calculated with 52 TB patients and 81 controls (ORD, HD and NTM). ROC (95% CI). PPV: positive predictive value; NPV: negative predictive value; A: accuracy. *p-value < 0.05.

*Humoral responses by quantitative IgA, IgG and IgM ELISA PPE59* - The pTB IgM class serum levels were not significantly different from HD^BR^ (p = 0.17), and the results remained inconclusive even after testing the pools of sera individually. They were not analysed further (Fig. 2C). There were, however, significant differences for IgG and IgA responses (p = 0.02), at the Ag concentration of 0.5 µg/mL and 1:50 serum dilution (Fig. 2A/B). For further analysis, these parametrisations were applied in the tests for all individuals.

After compilation of all ELISA results, patients’ clinical forms were opened, and the sera of TB participants showed generally higher IgA than IgG medians (p < 0.0001). The PPE59-IgA medians in pTB sera were highly significant compared to those of ORD/HD (p < 0.0001). Stratifying the population by geographic area of origin, medians of absorbance slightly decreased from Brazilian [1.039 (0.863-1.142)] through African [0.924 (0.515-1.277)] and Asian [0.890 (0.623-1.210)] to European pTB patients [0.770 (0.651-1.182), p = 0.89]. For eTB, Asians had a higher absorbance median [0.866 (0.532-1.155)], followed by Africans [0.609 (0.300-1.006)] and Europeans [0.593; (0.448-0.851); p = 0.31]; however, these medians were not significantly different from those in pTB (p > 0.0954). On the other hand, the PPE59-IgG medians elicited among pTB^BR^ [0.435 (0.301-0.662)] and Europeans [0.334 (0.207-0.443)], although lower compared to those obtained in IgA tests, were still above those for eTB^IT^ except Europeans [0.196 (0.159-0.380)], whose median was lower than those of ORD^BR^ [0.469 (0.351-0.630)] and HD^BR^ [0.241 (0.167-0.377)]. Contrary to IgA, NTM elicited a significant higher IgG median [0.904 (0.806-1.107)] compared with all other groups (p < 0.001) (Fig. 2D/E).

*Humoral responses by qualitative IgA and IgG ELISA PPE59* - Based on the differences observed in the quantitative analysis of the serologic tests, we further performed a ROC curve analysis to evaluate its potential diagnosis (Fig. 2F). The value of the area under the curve (AUC) was higher for IgA (0.823) than IgG (0.467) as well as the cut-off values of 1.030 (with LR of 21.81) and 0.732 (with LR of 1.043) respectively. The predicted sensitivity was variable, which denotes heterogeneity of the antibodies’ response based on the population tested. ELISA IgA sensitivity for pTB^BR^ was 53.3%, decreasing toward pTB^IT^ (38.2%) and eTB^IT^ (28%), with an overall detection of 42.7%, at a high specificity of 97.5%. The IgG sensitivity was 21.3%, 3%, and 0% respectively, with an overall detection of 9%, at lower specificity (83.1%). Contrary to IgA, IgG false positive was high among NTM. The overall accuracy of ELISA PPE59 IgA to detect TB was moderate (61.8%) with a higher positive predictive value (PPV = 96.5%) but a lower negative one (NPV = 47.9%), while both were lower for IgG. A combination of positive scores obtained for both isotypes tested did not improve accuracy, slightly increasing the sensitivity but jeopardising the specificity (Fig. 2G).

**Fig. 3:**
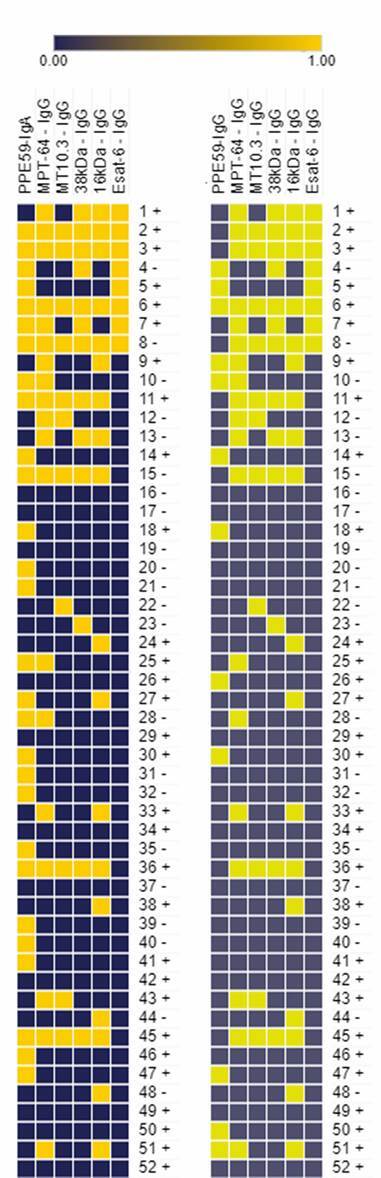
heat map of combinatory results of different antigens,(17) PPE59 and sputum smear microscopy (SM). Analysis of enzyme-linked immunosorbent assay (ELISA) IgG for ESAT-6, 16Kda, 38Kda, MT10.3, and MPT-64, IgA and IgG PPE59 in 52 serum samples from Brazilian patients with pulmonary tuberculosis and sputum SM results. Yellow indicates seropositivity; dark blue indicates seronegativity. + or -: sputum SM positive or negative. The heat map was generated using an online tool available at https://software.broad institute.org/morpheus/.

*TB detection value of IgA and IgG PPE59 combined with results of five other antigens previously tested* - Drawing upon the sera samples of Brazilian subjects previously tested with other mycobacterial antigens,^17^ we analysed the results in combination with those for the present PPE59 IgA and/or IgG. In the former study, lower ELISA IgG sensitivity was found for the single antigens ESAT-6 (22.5%), 16kDa (37.1%), MT10.3 (23.6%), MPT-64 (36%) or 38kDa (33.7%), with specificity ranging from 81.3% to 92%, which was below that of ELISA PPE59 IgA. However, the combination of positive results for PPE59 IgA plus ESAT-6 or 38kDa IgG slightly increased the sensitivity (55.8% or 59.6%), without jeopardising the specificity (91.4% or 95%) of the test. The combination of PPE59 IgA with 16kDa IgG achieved the highest sensitivity rate (71.1%). PPE59 alone identified 28/52 individuals while 16kDa identified only 18/52; however, 16kDa added positivity to 9/24 negative sera for PPE59, retaining a good specificity rate (88.9%). Notably, although 11 pTB^BR^ serum samples failed to react to any antigens (21%), 46.3% (19/41) did recognise at least one of the antigens, of which 68.4% (13/19) reacted to PPE59 only. Following up, 12 sera were reactive to two or three antigens, and ten sera were positive to four to all six antigens (Fig. 3A). The other IgA and/or IgG combinations did not yield better results; still, the use of 16kDa combined with PPE59-IgG achieved an improvement in the test, even if not superior to the response elicited by IgA [Fig. 3B, Supplementary data (Table)].

*ELISA performance compared to smear microscopy results* - While microscopy is not typically powerful enough when used as the sole screening and/or diagnostic method for TB, it can be done easily, rapidly and at a low cost. Thus, our approach was to combine two affordable methods to improve the detection of patients with active TB. The use of SM plus ELISA PPE59-IgA increased overall TB detection to 74%, compared to an SM detection rate of 70.8% (p = 0.01). ELISA PPE59 IgG produced no improvement, performing worse than SM alone (p = 0.69).

Within the stratified TB groups, there was a significant increase of positive SM/IgA results in group I, improving sensitivity to 80.7% (p = 0.01), with the use of serology detecting 12 SM-negative or untested pTB^BR^ patients (n = 3). In group II, sensitivity was only slightly increased, with four of the SM-negative eTB^IT^ (p = 0.26) and one pTB^IT^ (p = 0.82) detected. Combining SM, PPE59 IgA and 16kDa IgG ELISA results (available for Brazilians only), there was an improvement in sensitivity: out of the subjects not detected by PPE59 IgA (n = 10, of which 6 SM-negative and 4 SM-untested), 16kDa IgG recognised three subjects (two negative and one not tested with SM), bringing the sensitivity to 86.5% (p = 0.001) (Table II).

**TABLE II:** Sensitivity of acid-fast bacilli by Ziehl Neelsen-stained sputum smear microscopy (AFB-SM) combined with sensitivity of IgA, IgG enzyme-linked immunosorbent assay (ELISA) based on PPE59 and IgG 16kDa

	Number of positives (Total) % (95% CI)			
SM	pTB^BR^	pTB^IT^	eTB^IT^	All TB
Positive	30 (45) 66.6 (53 to 78.2)	39 (49) 79.5 (65.6 to 89.7)	11 (19) 57.9 (33.5 to 79.7)	80 (113) 70.8 (61.5 to 78.9)
Negative	15 (45) 33.3 (20 to 48.9)	10 (49) 20.4 (10.2 to 34.3)	8 (19) 42.1 (20.2 to 66.5)	33 (113) 29.2 (21 to 38.5)
Not done	7 (52)	5 (54)	6 (25)	18 (131)
SM vs SM-	*0.002*	*0.0001*	*0.337*	*0.0001*
SM^+^/IgA PPE 59	42 (52) 80.7 (67.4 to 90.3)	40 (54) 74 (60.3 to 85.0)	15(25) 60 (38.6 to 78.8)	97 (131) 74 (67.3 to 82.6)
SM vs SM/IgA	*0.01*	*0.82*	*0.26*	*0.02*
SM^+^/IgG PPE 59	32 (52) 61.5 (47 to 74.70)	39 (54) 72 (58.3 to 83.5)	11 (25) 44 (24.4 to 0.65.0)	82 (131) 62.6 (53.72 to 70.89)
SM vs SM/IgG	0.69	1.0	1.0	0.80
SM^+^/PPE59-IgA/ IgG-16kDa	45 (52) 86.5 (76.2 to 93.5)	NA	NA	NA
SM vs SM/IgA/IgG	0.001			

χ^2^: Pearson’s chi-square, with Yates’s correction; CI: confidence interval; NA: data not available.

*Uni- and multivariate analysis based on IgA and IgG ELISA PPE59* - Table III presents the association of PPE59 reactivity with the clinical and demographic characteristics of group I patients. Univariate analysis found PPE59 IgA positivity was associated with being male, alcohol and tobacco user, TST reactor, having positive sputum SM and abnormal CXR (p < 0.003). On the other hand, patients aged ≥ 40 years were more likely to have positive IgG results (OR = 2.8; 95%CI: 1.0-7.9; p = 0.04) as well as alcohol users (OR = 3.2; 95% CI: 1.1-8.8; p = 0.02) and those with CXR findings suggestive of high probability of TB (OR = 3.6; 95%CI 1.3-9.9; p = 0.0001). In contrast, in the multivariate model, the variables independently associated with a positive PPE59 IgA result were male (OR = 2.9; 95% CI 1.0-8.0; p = 0.03), alcohol user (OR = 2.7; 95% CI: 1.0-7.2; p = 0.04), and having CXR findings highly suggestive of TB (OR = 27.8; 95% CI: 2.7-281.2; p = 0.0001). Alcohol use remained independently associated with PPE59 IgG positivity (OR = 3.0; 95% CI 0.9-9.6; p = 0.05). In group II of the study, there were no statistically significant associations between clinical and laboratorial variables and positivity for either IgA or IgG PP59 ELISA (p > 0.30).

**TABLE III:** Multivariate and univariate analysis of demographic, clinical and laboratorial characteristics and positivity to IgA and IgG PP59 enzyme-linked immunosorbent assay (ELISA) in Brazilian pulmonary tuberculosis patients versus control cohort of patients with other respiratory disorders and healthy donors

	Univariate analysis				Multivariate analysis*			
	Study group I (CI 95%)							
Factors	IgA	p-value	IgG	p-value	IgA	p-value	IgG	p-value
Male	3.8 (1.4-9.7)	0.00	1.3 (0.5-3.4)	0.55	2.9 (1.0-8.0)	0.03	1.0 (0.3-3.0)	0.98
Female	1		1		1			
Age ≥ 40	1.1 (0.5-2.7)	0.66	2.8 (1.0-7.9)	0.04	1.1 (0.3-4.2)	0.81	3.6 (0.8-15.9)	0.08
Age ≤ 40	1		1		1		1	
Alcohol user	3.9 (1.5-9.7)	0.00	3.2 (1.1-8.8)	0.02	2.7 (1.0-7.2)	0.04	3.0 (0.9-9.6)	0.05
No alcohol user	1		1		1		1	
CXR high	5.5 (2.2-13.4)	0.00	3.6 (1.3-9.9)	0.00	27.8 (2.7-281.2)	0.00	5.4 (0.6-43.8)	0.11
CXR intermediary	22.7 (4.6-111.9)	0.00	1.4 (0.3-5.8)	0.59	9.5 (0.6-132)	0.09	1.0 (0-18.4)	0.99
CXR normal	1		1		1		1	

*Logistic regression analysis of demographic characteristic associated with PPE59 ELISA IgA positivity among Brazilian pulmonary tuberculosis patients (n = 52); CI: confidence interval; CXR: chest X-ray with changes of high and intermediate probability of tuberculosis.

## DISCUSSION

Several mycobacterial antigens have demonstrated potential for TB diagnostic testing over decades of studies including the use of multiple antigens.^21^ Broger et al. tested 57 Mtb antigens in field-based multiplexed serological assays against a reference assay based on 132 antigens to screen patients with suspected TB symptoms over two continents, finding that IgG performed similarly at three- or multiple-antigen tests with 35% sensitivity and 90% specificity.^21^ ^,^ ^22^ By contrast, single-antigen tests in our study achieved an overall 42.7% detection rate, attaining superior performance for IgA biomarker compared to IgG at higher specificity (97.5%), even comparing the samples of TB patients by geographic area and clinical forms of TB. The sensitivity rate improvement in ELISA IgA results was noteworthy when combinations of PPE59 IgA plus ESAT-6 or 38kDa IgG were analysed, maintaining better or similar specificity to those using multiple antigens.^21^ The 2017 study found the median reactivity levels varied between samples from Vietnamese and Peruvian subjects. Similarly, absorbance levels in our study were higher for the Brazilian cohort, decreasing toward European subjects but not achieving statistical significance other than for IgG. It should be noted that the lack of proper control from a non-endemic area may limit the interpretation of our results. Another possible explanation for the median variability is the fact that the mean time elapsed between the onset of symptoms and diagnosis may be longer than 90 days in Brazil, which may lead to a more invasive disease progression as suggested by the frequency of CXR changes of high TB probability. By comparison, TB diagnosis takes place faster in Italy, where CXR changes tend to be less extensive, and treatment begins before the patient starts bleeding. As reported in other studies, multi-cocktails of antigens or their peptides remain a good strategy to maximise accuracy in a diagnostic test; the biggest challenge lies in finding the best antigens and isotype combination.^15^ ^,^ ^22^ Note that a combination of PPE59 IgA plus 16kda IgG brings sensitivity to 74%, and adding the results of other rapid tests (SM = 66%) raises it to 86.5% (at 88.9 % specificity), making this combination a potential target product profile for composing rapid diagnosis tests in low-income areas highly burdened with TB. This assumption relies on the fact that ELISA test easily adapts for a point-of-care platform. However, our data is based on Brazilian subjects only, and requires further evaluation.

In addition, the sensitivity of SM was significantly variable (53-78.2%, p = 0.002, Table II). Although ELISA PPE59 was not powerful enough to equally diagnose TB, serology improved case finding, mainly among negative SM cases of pulmonary or extrapulmonary clinical form, based on IgA PPE 59 single antigen or IgG multiple antigens. As an explanation, the production of antibodies may not depend on the bacillary load and rather at the site of infection. Therefore, serology may be an additional rapid tool for detecting TB cases, and an advantage of using PPE59 IgA is its high specificity when dealing with populations of low-resource areas.

The highest IgA PPE59 absorbance (close to the cut-off) was found among COPD and smoking patients in the control group. COPD may be a risk factor for the development of pulmonary TB, and both diseases share common risk factors, such as smoking and low socioeconomic status.^23^ Moreover, the two ORD individuals who were positive for PPE59 IgA had a history of recent contact with active TB patients; therefore, we cannot rule out the possibility of Mtb infection, potentially progressing toward active TB. Contrary to PPE59 IgA, IgG cross-reactivity was higher among NTM sera; corroborating the fact that, in addition to antigens, the choice of isotypes is important, as not all antibody isotype responses are appropriate biomarkers for clinical TB.

To date, the actual function of PPE59 protein in metabolism and its evolutionary role in Mtb is still unknown. Indeed, it has been demonstrated by past studies to induce cell-mediated response by IFN-γ and interleukin.^14^ ^,^ ^24^ ^,^ ^25^ ^,^ ^26^ On the other hand, our study found moderate frequency of IgA reactivity and lower IgG in the sera of patients with TB. In a study carried out in China and India, IgG recognised PPE57 (*rv3425*) and PPE17 (*rv1168*) proteins, respectively, with high sensitivity and specificity rates (100%) even in extrapulmonary TB cases.^12^ ^,^ ^27^ But then again, the control group was smaller (n = 20) and did not comprise ORD patients, unlike in our study. These results may also suggest that PPE59 and PPE57 bacteria expression may change under different host conditions since identification occurs by different antibody isotypes.

Studies of different antigens have shown an increase of IgG ESAT6/16kDa/HBHA in active TB (declining after treatment) and in latently infected contact, suggesting an association of specific antibodies with the bacillary load.^28^ While IgA-HrpA levels correlated significantly with active TB, significant association of IgA-16kDa/ESAT6 with the nutritional status of the participants was described. Others reported higher response from IgA anti-mycobacterial antigens HrpA and MDP1 among controls than in TB patients, suggesting that different stress on bacterium and host modulates proteomic expression, and the IgA immune response may be protective.^29^ ^,^ ^30^ Williams et al.^31^ reported IgA against the 16 kDa α-crystallin mediated some protection against TB, as evidenced by reduced mycobacterial burden in the lungs. However, we found an association of IgA PPE59 reactivity with high TB probability CXR alterations. Considering that most TB cases in Brazil are diagnosed at advanced disease development stages during self-referred patient visits to the health care, it could be speculated that PPE59 is more expressed at a later phase of the clinical evolution of TB disease. On the other hand, alcohol-using TB patients (among both Brazilian and Italian/immigrant subjects) are ≥ 2.7 times more likely to test positive for PPE59 IgA or IgG. Similar results have been observed for 38kDa and MPT-64 IgG responses tested previously in the same Brazilian population.^17^ Studies^32^ ^,^ ^33^ assessing quality of life among individuals with pulmonary TB in Africa have demonstrated the harmful effect of alcohol use among TB patients, which negatively influences the immune system when in excess. However, the mechanism involved remains to be clarified. Our study was not designed to address the effect of alcohol in the humoral response of TB patients; specific studies are needed to examine such serological considerations. Moreover, prior treatment may have introduced a bias toward lower positive IgA PPE59 frequency in IT patients. Other recent studies have reported low response from LppZ-specific IgA after 2 months of anti-TB treatment,^30^ as well as for anti-HBHA IgA and IgG and IgA against Rv2031, whereas anti-LAM IgA and IgG increased following chemotherapy.^34^ However, our study was not designed to follow up on treated patients. There were no other associations of demographic or clinical characteristic with antibody positivity among Italian/immigrants, especially for IgG, due to the low number of individuals who tested positive.

The main function of IgA is to exert an effector action on the mucosal immune system and act as an important defence against pathogens that invade the host via their mucosa, such as Mtb.^15^ ^,^ ^31^ IgA serves a variety of protective functions such as interfering with pathogen adherence to mucosal cells (immune exclusion), causing intracellular neutralisation and excretion of the pathogen. Its monomeric form, present in the serum, has a complementary action, eliminating pathogens that have crossed the mucosal barrier and still interact with the Fc receptors of cytotoxic cell-dependent antibodies.^35^ Therefore, the presence of detectable PPE59 IgA in TB patient sera may fulfil a protective action, containing the bacilli that escaped the mucosal barrier, whose immune response best controls bacillary multiplication.

This study has several limitations, including a heterogeneity of specific treatments between study groups, an unavailability of proper controls in group II, insufficient TB^IT^ sera samples to allow for an evaluation of other antigens, and a lack of follow-up immune response from treated patients.

In conclusion**,** tests using PPE59 at high specificity elicited moderate frequency of IgA, lower IgG, and no IgM responses among TB patients. Although evaluating PPE59 IgA alone does not allow for accurate enough results, this biomarker may be an additional rapid tool for detecting symptomatic suspected TB patients in yet-unexplored combination with 16kDa IgG and SM tests, and deserves prospective evaluation in highly TB-burdened, low-resource areas. Moreover, we found a strong association of PPE59 IgA reactivity with the presence of CXR alterations of high TB probability (p = 0.001) and alcohol use (p = 0.04), which warrants further investigation.
