# Supplementary material for: PPE59 antibodies in tuberculous patients and potential use for diagnosis when assayed with other rapid biomarkers
Source: Mem Inst Oswaldo Cruz. 2024 Sep 16;119:e230183. doi: 10.1590/0074-02760230183 (PMC11404981; doi:10.1590/0074-02760230183)
Supplement: Supplementary file 1 [file 1678-8060-mioc-119-e230183-s.pdf]

TABLE

Sensitivity and specificity of IgA and/or IgG enzyme-linked immunosorbent assay (ELISA) based on PPE59 antigen and combinatory analysis of IgG ELISA results of different antigens (ESAT-6, 16Kda, 38Kda, MT10.3, and MPT-64) (17) among serum samples of Brazilian subjects (pTB^{BR} and $NoTB^{BR}$)

	IgA		IgG	
	pTB^{BR} (n = 52)	NoTB (n = 81)	pTB^{BR} (n = 47)	NoTB (n = 77)
PPE59 combination with IgG against other mycobacterial antigens	Sensitivity n (%) [95% CI]	Specificity n (%) [95% CI]	Sensitivity n (%) [95% CI]	Specificity n (%) [95% CI]
PPE59 + ESAT-6	29 (55.8) [42.3 to 68.4]	4 (95) [88 to 98]	17 (36.1) [23.8 to 50.5]	12 (84.4) [74.7 to 90.8]
PPE59 + 16kDa	37 (71.1) [57.7 to 81.6]	9 (88.9) [80.2 to 94]	25 (53.1) [39.2 to 66.7]	17 (78) [67.4 to 85.7]
PPE59 + 38kDa	31 (59.6) [46.1 to 71.8]	7 (91.3) [83.2 to 95.7]	22 (46.8) [33.3 to 60.7]	13 (83.1) [73.2 to 89.9]
PPE59 + MT10.3	31 (59.6) [46.1 to 71.8]	8 (90.1) [81.7 to 95]	22 (46.8) [33.3 to 60.7]	17 (78) [67.4 to 85.7]
PPE59 + MPT64	35 (67.3) [53.7 to 78.5]	8 (90.1) [81.7 to 95]	27 (52) [38.7 to 65]	17 (78) [67.4 to 85.7]
PPE59 + ESAT-6 +38kDa	31 (59.6) [46.1 to 71.8]	9 (88.9) [80.2 to 94]	21 (40.4) [28.1 to 54]	13 (83.1) [73.2 to 89.9]
PPE59 + All antigens	41 (78.8) [65.9 to 87.7]	18 (77.8) [67.6 to 85.5]	34 (65.4) [51.8 to 77]	24 (68.8) [58 to 78]
PPE59 IgA/IgG + all other IgG antigens	43 (82.7) [70.3 to 90.6]	25 (52.1) [36.3 to 65.5]		

NoTB: comprising patients with other respiratory disorders, including those infected with non-tuberculous mycobacteria, and asymptomatic donors; pTB: confirmed pulmonary tuberculosis.

CC BY
